# The Effect of Enteric-Derived Lipopolysaccharides on Obesity

**DOI:** 10.3390/ijms25084305

**Published:** 2024-04-13

**Authors:** Kai Wang, Weiwen Lai, Tianqi Min, Jintao Wei, Yan Bai, Hua Cao, Jiao Guo, Zhengquan Su

**Affiliations:** 1 Guangdong Provincial University Engineering Technology Research Center of Natural Products and Drugs, Guangdong Pharmaceutical University, Guangzhou 510006, China; wk972023826@163.com (K.W.); laiweiwengy@163.com (W.L.); 15618039319@163.com (T.M.); starwjt@163.com (J.W.); 2 Guangdong Metabolic Disease Research Center of Integrated Chinese and Western Medicine, Guangdong Pharmaceutical University, Guangzhou 510006, China; 3 School of Public Health, Guangdong Pharmaceutical University, Guangzhou 510310, China; angell_bai@163.com; 4 School of Chemistry and Chemical Engineering, Guangdong Pharmaceutical University, Zhongshan 528458, China; caohua@gdpu.edu.cn

**Keywords:** lipopolysaccharide, endotoxin, intestinal flora, obesity, targeted therapy

## Abstract

Endotoxin is a general term for toxic substances in Gram-negative bacteria, whose damaging effects are mainly derived from the lipopolysaccharides (LPS) in the cell walls of Gram-negative bacteria, and is a strong pyrogen. Obesity is a chronic, low-grade inflammatory condition, and LPS are thought to trigger and exacerbate it. The gut flora is the largest source of LPS in the body, and it is increasingly believed that altered intestinal microorganisms can play an essential role in the pathology of different diseases. Today, the complex axis linking gut flora to inflammatory states and adiposity has not been well elucidated. This review summarises the evidence for an interconnection between LPS, obesity, and gut flora, further expanding our understanding of LPS as a mediator of low-grade inflammatory disease and contributing to lessening the effects of obesity and related metabolic disorders. As well as providing targets associated with LPS, obesity, and gut flora, it is hoped that interventions that combine targets with gut flora address the individual differences in gut flora treatment.

## 1. Introduction

Endotoxins were first discovered and named by Richard Pfeiffer in the 18th century when he was studying the relationship between cholera and Vibrio in guinea pigs [1]. Previous studies have shown that LPS plays a key signalling role in the development of infections, inflammation, sepsis, and multi-organ failure [2]. LPS are very stable and familiar; it takes 3 h of heating at 180 °C to destroy them [3,4]. LPS are found in the outer membranes of Gram-negative bacteria, which colonise the human mouth and intestine. And we all consume different foods daily, significantly increasing the body’s exposure to LPS. And all these other foods can have some effect on the general intestinal flora [5]. Many recent studies have shown that LPS are inextricably linked to obesity [6,7,8,9]. Research has shown that at least some of the inflammation associated with obesity originates in the gastrointestinal tract [10]. Diet-induced obesity is associated with changes in the composition of the intestinal microbiota and intestinal permeability, leading to the increased production and transport of pro-inflammatory bacterial products, such as LPS, in the gut [11]. Nowadays, more than a quarter of the world’s population is affected by obesity, which has reached epidemic proportions and has become a social problem that cannot be ignored [12].

The intestinal flora has been shown to play a crucial regulatory part in human metabolism and can even be seen as a complex metabolic organ of the body [13]. If the problem of obesity and the dangers of LPS could be addressed simultaneously through the gut flora, the burden on global healthcare resources could be significantly reduced, and the health of all humans could be improved. This paper is therefore based on a closed-loop hypothesis about the impact of LPS on obesity. The dysbiosis and apoptosis of the intestinal flora produce large amounts of LPS, LPS induce inflammation and contribute to the progression of obesity, and finally, obesity, in turn, affects the structure and metabolic profile of the intestinal flora.

## 2. Lipopolysaccharide

### 2.1. Structure of LPS

**Figure 1:**
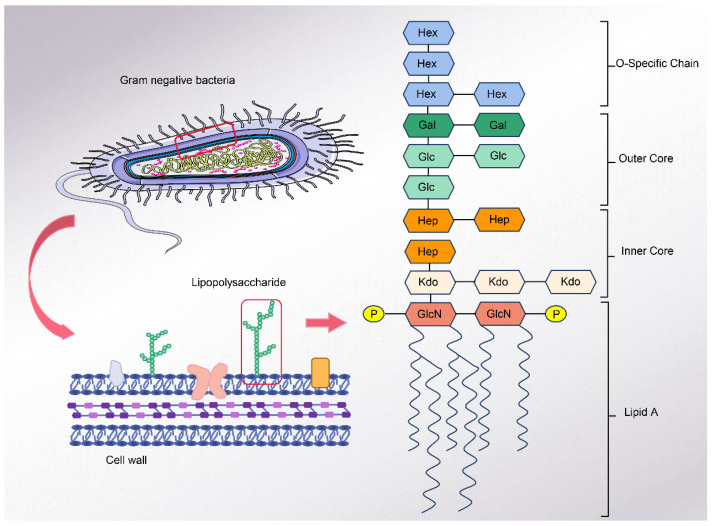
LPS structure. GlcN: glucosamine. P: phosphate group. Kdo: 3-deoxy-D-manno-octulosonic acid. Hep: glycero mannoheptose. Glc: glucose. Gal: galactose. Hex: hexose.

The structure of LPS is usually divided into three regions: the O-specific chain, which consists of hydrophilic polysaccharides; the core region; and the hydrophobic lipid component, generally referred to as lipid A [Figure 1]. The core region is further divided into an inner core, covalently linked to the lipid A portion, and an outer core, connected to the O-specific chain portion [14,15,16]. The O-specific chain is complex and variable, while lipid A is the least changed part. Not all bacterial strains have a complete O-specific chain, outer core, and inner core part, but they all contain lipid A [17,18]. An example is the deep rough mutant of Haemophilus influenzae (I-69 Rd−/b+), a bacterium whose lipopolysaccharide structure consists only of lipid A and a phosphorylated dOclA residue but which can still survive and reproduce normally [19]. Lipid A consists of hydrophilic heteropolysaccharides and covalently bound lipid components [20,21,22]. The specific binding of lipid A to the recognition molecule is mainly mediated by its hydrophilic backbone (phosphorylated-d-glucosaminoglycan) [23]. It has been found that synthetic *E. coli* lipid A has the same lethal toxicity, thermogenic, and induction of local Shwartzman reaction properties compared to natural lipid A, and they cannot be distinguished from each other in terms of various parameters [24]. It has also been found that synthetic lipid A compounds lack cell-stimulating activity [23]. However, all can indicate that the toxicity and immune activity of LPS is mainly determined by lipid A. The structure of inactivated LPS lipid A is altered, e.g., lipid A with a laminar structure, which has no LPS activity and lacks cytokine induction, and lipid A with a non-laminar inverted design, which exhibits intact toxicity both in vitro and in vivo. This indicates that specific structures and conformations are also required for the complete expression of the activity [25]. In addition, the negative charge present in the LPS molecule is an essential factor affecting the biological activity of LPS [26].

### 2.2. Mechanism of Inflammation Induced by LPS

After entry into the organism and bacterial death, the lipopolysaccharide is released. It forms a multimer that enters the circulation and acts through the positively charged arginine and lysine residues in the N-terminal portion of lipid A between amino acids 91 and 108, binding to the lipopolysaccharide binding protein (LBP) [27,28]. LBP promotes the monomerisation of LPS and synergises with cell membrane-bound CD14 to transport LPS. CD14 transports the complex of LPS and LBP by shedding cell-bound receptor molecules to bind at toll-like receptor proteins [29,30]. Toll-like receptors (TLRs) act as sentinels of the immune system, sensing pathogen-associated molecular patterns (PAMPs), with TLR2 and TLR4 receptors sensing LPS [31,32]. One study used TLR4 knockout mice as opposed to mice hyporesponsive to LPS; both showed the same degree of LPS hyporesponsiveness [33]. Other studies also found no significant effect of commonly expressed TLR2 on LPS signalling in mice lacking TLR4 [34]. These suggest that the TLR4 receptor protein primarily mediates the LPS-induced inflammatory response in the organism. Upon binding to the TLR4 receptor, the junction protein MyD88, which acts as a universal adapter for the activation of the inflammatory pathway, is recruited to the intracellular region of TLR4 (TIR structural domain) and transduces the signal from extracellular to intracellular [35,36]. MyD88 dimerises and recruits IL-1 receptor-associated kinase (IRAK) via the death effector domain; IRAK undergoes autophosphorylation and activation, and the activated IRAK binds to the tumour factor-associated receptor (TRAF6) [37,38,39]. The bound TRAF6 is ubiquitinated by the ubiquitin ligases Ubc13 and Uev1A, which activate TAK1 (transforming growth factor-activated kinase 1), which in turn phosphorylates a complex of IKK (IκB kinase, the inhibitor protein of NF-κB), consisting of two catalytically active kinases, IKKα and IKKβ, and the regulatory subunit IKKγ (NEMO) [40,41]. The NF-κB protein is generally formed as a homo/heterodimer from p50 and p65. It is inactivated in the cytoplasm of cells by binding to the inhibitor protein IkB to form a trimeric complex. IKK (the inhibitor of kappa B kinase) dissociates IκB from the trimeric complex formed with NF-κB, which is then degraded by phosphorylation by the corresponding protease [42,43]. The restored NF-κB dimer exposes its nuclear localisation sequence and rapidly enters the nucleus from the cytoplasm to bind to specific sequences in the nuclear DNA, initiating the transcription of relevant inflammatory factor target genes and increasing the synthesis and expression of various cytokines such as TNF-α, IL-1, IL-6, and IL-8 at the gene level [44,45].

In addition, TRAF6 interacts directly with ECSIT (an evolutionarily conserved signal transduction intermediate in the toll pathway) and provides a link to MEKK-1 (a kinase of MAPK kinase) [46]. This is followed by the activation of MEK (MAPK kinase) and MAPK (mitogen-activated protein kinase), which is the tertiary signalling process of the MAPK pathway. The MAPK signalling pathway is composed of three subunits: extracellular signal-regulated kinase (ERK), p38 mitogen-activated protein kinase (p38), and c-Jun amino-terminal kinase (JNK), which are activated in turn [47]. The final result is the activation of Jun and Fos, the AP-1 family of transcription factors. At the same time, TAK1 is also a member of the MAPKKK family, and the activation of TAK1 leads to the phosphorylation of MKK4, MKK6, and MKK7, which in turn activates members of the MAPK family [48]. This mediates the activation of AP-1 (transcription activator protein 1), which plays a vital role in cell growth, apoptosis, the inflammatory response, and oxidative stress [49].

Another pathway that does not need to go through MyD88 is called the MyD88 non-dependent pathway, also known as the TRIF-dependent pathway. This pathway is initiated by TRAM and TRIF (splice protein in the TLR4 signalling pathway). TLR4 signalling via the TRIF bridging protein leads to the K63-linked polyubiquitination of TRAF3, which results in the activation of TANK-binding kinase 1 (TBK1) and IκB kinase-ε (IKKε), which in turn phosphorylates interferon regulatory factor 3 (IRF3) and interferon regulatory factor 7 (IRF7), forming a dimer and leading to translocation to the nucleus and finally to the activation of inflammatory factor expression. In addition, TANK-binding kinase 1 (TBK1) also acts directly on IκB, leading to the activation of the NF-kB protein [50,51,52].

**Figure 2:**
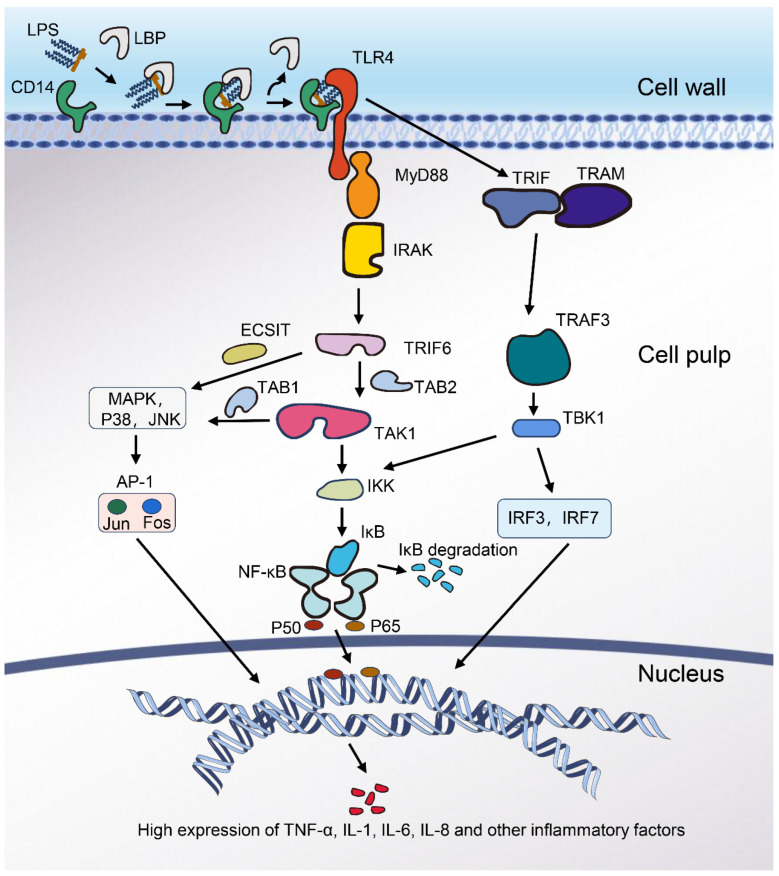
LPS inflammatory mechanisms. LPS: (lipopolysaccharide); LBP: (lipopolysaccharide binding protein); CD14: (cluster of differentiation 14); TLR4: (toll-like receptor 4); MyD88: (myeloid differentiation factor 88); IRAK: (IL-1 receptor-associated kinase); TRIF: (toll/interleukin-1 receptor); TRAM: (tRIF-related adaptor molecule); TLR: (toll-like receptor); TRAF: (tumour necrosis factor receptor-associated factor); IKK: (IκB kinase); TAK1: (transforming growth factor-β-activating kinase); ECSIT: (evolutionarily conserved signalling intermediate in toll pathway); IRF: (IFN regulatory factor); TAB: (TAK1 binding protein); IκB: (inhibitor of NF-κB); P50 and P65: (NF-κB family member factor); TBK1: (TANK-binding kinase); MAPK: (mitogen-activated protein kinase); p38: (p38 mitogen-activated protein kinase); JNK: (c-Jun amino-terminal kinase); AP-1: (transcription activator protein 1); NF-kB: (nuclear factor kappa-B); IL: (interleukin); TNF-α: (tumour necrosis factor-alpha).

The LPS-mediated inflammatory signalling pathways described above show a degree of crosstalk between the MAPK pathway and the NF-κB pathway, as well as between the MyD88-dependent and TRIF-dependent pathways [Figure 2]. This induces an inflammatory response in the organism, leading to damage [53].

## 3. LPS and Obesity

Metabolic endotoxaemia, defined as a two- to three-fold increase in circulating LPS levels, has been proposed as the initiating factor for obesity-related metabolic dysfunction [54]. Intestinal flora has long been shown to induce metabolic endotoxaemia by releasing endotoxins, promoting systemic hypo-inflammation and insulin resistance [55,56]. As a result, increased LPS can lead to inflammation, weight gain, and diabetes.

Obesity is a low-grade chronic inflammatory condition coordinated by metabolic cells [57,58]. Adipose tissue (AT) volume in obese individuals is associated with increased levels of pro-inflammatory cytokines (e.g., TNF-α, IL-1, and IL-6) and acute phase proteins [59,60]. The most notable biological activity of LPS is the triggering of systemic and local inflammation in the body, and the resulting inflammation is more or less a further enhancement of the degree of obesity, for example, in rodent models of obesity, where intestinal microbial LPS are responsible for the enhanced pro-inflammatory environment [8]. Plasma phospholipid transfer protein (PLTP) is an immunomodulatory transfer protein in plasma, which can cooperate with lipoprotein to neutralise the toxicity of LPS [61]. In the case of a high-fat diet, mice that did not express PLTP activity gained 21.7% more weight than mice that expressed it normally, and the lipid content of visceral adipose tissue increased, the size of adipocytes increased, and inflammatory factors and circulating LPS also increased [62]. Studies have shown that human adipose tissue and inflammation are closely related to LPS levels. By detecting plasma LPS, adipose tissue, and inflammation-related factors in obese subjects, it is found that LPS can affect the adipose tissue function of obese people by affecting the gene expression of lipogenesis and increasing the gene expression of inflammatory factors [63]. LPS from the intestinal tract can inhibit the synthesis of mitochondria, damage the browning function of adipocytes, lead to the dysfunction of adipocytes, and greatly increase the risk of obesity-related diseases [64]. LPS can induce adipocytes to produce inflammatory factors at different stages of adipocyte differentiation, and at the same time, it can also lead to the increase in iron accumulation in adipocytes, destroy iron homeostasis, and then affect the metabolism of the whole body [65]. Also, obesity is associated with increased intestinal permeability in humans [66]. More precisely, it is intestinal permeability that is positively correlated with visceral obesity and liver fat content [67]. By comparing obese mice with non-obese mice, Paola Brun [68] found that obese mice exhibited increased intestinal permeability, a significantly altered distribution of occludin and ZO-1 in the intestinal mucosa, and elevated levels of both inflammatory factors and portal LPS. This supports the notion that mucosal barrier damage is increased in the presence of obesity. A similar situation will occur when LPS is given to obese mice induced by a high-fat diet, which will aggravate LPS-induced small intestinal injury, oxidative stress, and the decrease in the tight junction (the expression levels of ZO-1 and occludin are reduced) [69]. Intestinal permeability affects changes in the intestinal flora. For example, the induction of intestinal flora imbalance by LPS exhibits more pronounced intestinal damage and an increased expression of β-defensins and Muc2. In contrast, faecal transplantation has been shown to reconstitute the intestinal flora, down-regulate β-defensin and Muc2 expression, and reduce intestinal damage [70]. And continued exposure to LPS and LPS-induced soluble pro-inflammatory mediators may further exacerbate inflammation and obesity [71,72]. So crosstalk between gut flora, obesity, and LPS is a logical scenario.

## 4. Obesity and Intestinal Flora

At first, it was thought that the composition of intestinal flora might be largely determined by genes. Later, some studies denied this point. Researchers analysed the faecal microbiota of identical twins and fraternal twins and found that although there were some similarities between them, there was no significant difference between the microbiota of identical twins and fraternal twins [73]. This shows that the intestinal flora is more affected by acquired factors. In order to further clarify the relationship between obesity and intestinal flora, a study compared the differences in intestinal flora composition between obese and non-obese people. The results showed that the proportion of Bacteroides in obese people was lower, and the proportion of *Firmicutes* was higher [74]. This phenomenon is also reflected in mice. Genetically obese (ob/ob) mice have 50% less *Bacteroidetes* than lean mice. Fortunately, this phenomenon can be improved through diet [75]. However, it should also be noted that, possibly due to differences in diet, genetic background, living environment, overall health status, and measurement methods, some studies have observed a different gut flora profile in obese people: *Firmicutes* are lower, and *Bacteroidetes* are higher [76]. Even if they are both obese people, the intestinal flora of each person has diversity and specificity. Specifically, *Firmicutes*, *Fusobacteria*, *Proteobacteria*, and *Lactobacillus reuteri* are more numerous in the intestines of obese people, while *Bacteroidetes*, *Faecalibacterium prausnitzii*, and *Akkermansia muciniphila* are less numerous [77,78,79]. Intestinal flora in obese people can absorb more energy from food, further regulating host metabolism and weight changes in the host [80]. Compared with healthy people, the intestinal flora of different obese people is more similar, and the intestinal microbial diversity is lower [81,82]. For obesity, we should not only study the impact of intestinal flora but also pay attention to their metabolites. Active intestinal flora will produce a large number of physiologically active substances, including short-chain fatty acids, vitamins, and healthy substances, as well as potentially harmful substances, such as LPS. Circulating LPS levels were found to be generally low in healthy people, directly related to food intake, while circulating LPS were usually higher in obese people [83]. A doubling of plasma LPS concentrations and a two- to five-fold increase in the expression of TNF-α, IL-6, and IL-8 in cells were significantly observed in a group of obese women who were pregnant [84]. Epidemiological studies have also shown that plasma concentrations of LPS, LBPs, and pro-inflammatory cytokines are higher in obese than non-obese populations [85]. The study also found that animals injected with LPS had hypokinetic shock and significantly decreased arterial pressure. However, the injection of LPS into obese pigs resulted in more severe haemodynamic failure and more obvious microcirculation dysfunction [86]. Changes in intestinal flora affect body weight, reducing circulating LBPs and LPS following fat loss [8]. LBP can therefore be seen as an inflammatory marker associated with obesity, and further evidence is needed as to whether intestinal LPS can influence obesity through the intestinal flora.

## 5. LPS and Intestinal Flora

The intestinal flora is already a non-negligible factor in the pathogenesis of obesity. For example, previous studies have shown that mice with a sterile gastrointestinal tract resist high-fat diet-induced obesity [87,88]. Among these, Gram-negative bacteria in the intestinal flora may be vital to developing obesity. According to previous descriptions, the presence of higher numbers of *Fusobacteria* and *Proteobacteria* in obese people are all Gram-negative bacteria. A multi-ethnic cohort study explored the relationship between diet and obesity by analysing intestinal flora, the energy-adjusted dietary inflammatory index (E-DII), total fat mass, and visceral adipose tissue. It is found that intestinal microorganisms and LPS mediate the relationship between E-DII and obesity. LPS, Flavonifractor, gnavus, and Tyzzerella played a significant role in the whole research results, while Flavonifractor and Tyzzerella were all Gram-negative bacteria [89]. To investigate the effect of a single LPS producer on obesity, Fei, N [90] obtained a clinical isolate (B29) from volunteers’ faecal samples, identified it as *Enterobacter cloacae* (Gram-negative) by biochemical tests and 16S ribosomal RNA genes, and horseshoe crab deformed cell lysate tests showed that the lipopolysaccharide of B29 had endotoxic solid activity. In a high-fat diet (HFD), germ-free C57BL/6J mice with B29 (HFD + B29) induced the full development of obesity and more fat at epididymal, mesenteric, subcutaneous inguinal, and retroperitoneal fat pad masses in (HFD + B29) compared to germ-free C57BL/6J mice and Luria–Bertani medium (HFD + LB) and exhibited an increased serum LPS load and an increased expression of TNF-α, IL-1b, IL-6, and TLR4 pro-inflammatory genes. In addition, volunteers who provided faecal samples reduced their weight by adopting a diet consisting of whole grains, herbal foods, and prebiotics showed a decrease in *Enterobacter* spp. from 35% to 1.8% and improved most metabolic parameters to the normal range. A healthy foetus’s gastrointestinal tract is sterile, and it is only after birth that bacteria from the mother and the surrounding environment colonise the infant’s gastrointestinal tract. By injecting pregnant rats with LPS and euthanising the pups at 0, 3, and 7 days after a natural birth and collecting their intestinal tissues and faeces, the researchers found significant changes in the intestinal flora of the pups exposed to LPS prenatally and a significant increase in the number of *Aspergillus* spp., at 7 days after birth [91]. This indicates that LPS have the ability to independently affect the intestinal flora. Increased expressions of LPS levels, TLR4, TNF-α, and IL-18 were detected in mice consuming high-fat chow along with increased *E. coli* in the body [92]. Even when the same high-fat diet is consumed, the different components of the composition can have other effects on the organism [93].When mice were fed the same calories of lard and fish oil (rich in saturated and polyunsaturated lipids, respectively), a significant increase in the accumulation of CD45 cells as well as the activation of TLR2 and TLR4 in serum and a trend towards higher LPS levels were observed in white fat only in mice fed lard, suggesting that the lard diet promotes the greater influx of microbial factors into the body’s circulation. However, bacteriophages (*Aspergillus* spp., Gram-negative bacteria) increased in mice regardless of which oil was fed, suggesting that there may be a correlation between lipid intake and an increase in the number of Gram-negative bacteria. Next, the transplantation of the intestinal flora from mice fed fish oil into mice fed lard was found to counteract the obesity and inflammation of mice fed lard and a trend towards lower serum levels of LPS in mice. Finally, a comparison of non-obese mice, MyD88 receptor-deficient mice, and TRIF receptor-deficient mice revealed that mice deficient in MyD88 or TRIF were protected from lard-induced WAT inflammation and impaired metabolism and that saturated dietary lipids and gut microbiota interacted to induce WAT inflammation. This suggests that we can improve obesity and inflammation by altering the type of lipids consumed. Moreover, dietary saturated fatty acids may mediate impaired white fat inflammation and metabolism via the TRIF and MyD88 pathways, which overlap with the paths through which LPS-mediated inflammation and metabolism occur.

The amount of LPS and the differences in the nature and composition of the LPS derived from different microorganisms lead to differences in the levels of inflammation they activate [94,95]. For example, the injection of LPS from *E. coli* triggered LPS tolerance in non-obese diabetic mice and reduced the incidence of diabetes in these mice. These effects were not observed with LPS from *B. dorei* [96].

Even the relatively small number of Gram-negative bacteria in obese people has an essential influence on the development of obesity. *Akkermansia muciniphila*, a mucin-degrading bacterium of *Verrucomicrobia*, accounts for only 1% to 5% of the human intestinal microbiota. Its abundance is negatively correlated with body weight in mice and humans [97]. Higher circulating LPS levels inhibit adipose tissue differentiation and adipogenesis, leading to altered adipose tissue metabolism in obesity [98]. Therefore, it is speculated that *Akkermansia muciniphila* can restore intestinal barrier function and normalises metabolic endotoxaemia and adipose tissue metabolism. *Akkermansia muciniphila* counteracted diet-induced barrier dysfunction in the colonic mucosa during obesity [99], mainly by affecting the thickness of the inner mucus layer, which was 46% thinner in mice fed a high-fat diet, and this change was counteracted by *Akkermansia muciniphila* treatment. At the same time, *Akkermansia muciniphila* treatment normalised diet-induced metabolic endotoxaemia, obesity, and the adipose tissue marker CD11c [100] and reduced body weight and improved body composition (i.e., fat mass/lean body mass ratio) without an altered food intake. This supports the idea that increased intestinal permeability leads to metabolic endotoxaemia. Mice fed a high-fat diet exhibited higher portal LPS concentrations than mice fed a regular diet. Treatment with *Akkermansia muciniphila* restored LPS concentrations to levels found in the regular diet group. Moreover, the LPS from *Akkermansia muciniphila* is structurally different from *E. coli* and is not a potent TLR4 agonist. The specific activation of cells expressing TLR2 but not TLR5, TLR9, or NOD2 receptors by *Akkermansia muciniphila* was observed in the experiment [101]. The administration of ApoE-deficient (Apoe/−) mice to a Western diet and the oral administration of *Akkermansia muciniphila* revealed a significant attenuation of metabolic endotoxaemia, reduced macrophage infiltration, and pro-inflammatory cytokine and chemokine expression. In contrast to normal food-fed mice, *Akkermansia muciniphila* mediated a reduction in circulating LPS levels by blocking LPS infiltration to protect the intestinal barrier through the induction of intestinal tight junction protein (ZO-1 and occludin) expression [102]. These results all point to a critical role for *Akkermansia muciniphila* (Gram-negative bacteria) in the physiopathology of obesity and metabolic inflammation.

## 6. LPS-Related Targets

**Figure 3:**
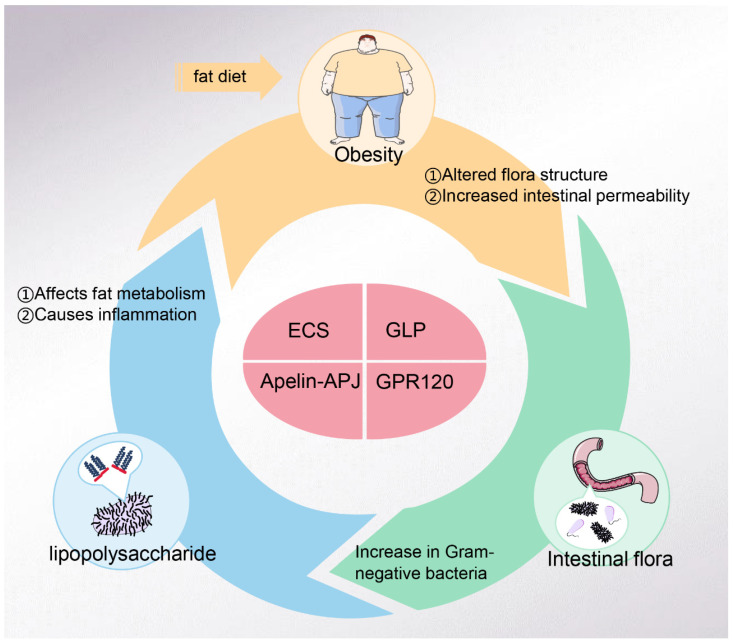
The relationship between LPS, obesity, and intestinal flora. ECS: endocannabinoid system; GLP: glucagon-like peptide; Apelin–APJ: Apelin is a regulatory peptide and a ligand of the G protein-coupled receptor (APJ); GPR120: G protein-coupled receptor 120.

LPS of various microbial origins enter the bloodstream through the intestinal tract and cause low levels of inflammation throughout the body. LPS have been identified as a trigger for disease in various chronic diseases [6,103]. Therefore, understanding the targets associated with LPS is essential for preventing and treating LPS-induced diseases [Figure 3, Table 1].

### 6.1. ECS (Endocannabinoid System)

The ECS consists of endogenous cannabinoids (eCBs), cannabinoid receptors, and various enzymes responsible for the synthesis and degradation of endogenous cannabinoids. Of these, CB1 and CB2 are the most typical cannabinoid receptors, 2-AG (2-arachidonoylglycerol) and AEA (N-arachidonoyl ethanolamine) are two of the most widely studied endocannabinoids, and AEA and 2-AG are also agonists for CB1 and CB2 [121,122]. Obesity is characterised by changes in the gut microbiota and the development of low-grade inflammation and increased concentrations of endogenous cannabinoids. There was also a positive correlation between LPS and endogenous cannabinoid levels [123,124,125]. Several studies have shown an association between LPS and the ECS. The cannabinoid 1 receptor (CB1) can affect LPS-induced inflammation. An experiment comparing the expression of LPS-related proteins in the livers of mice in the blank, LPS, and LPS + CB1 antagonist groups found that MyD88 and p-NF-κB protein levels were significantly reduced in the LPS + CB1 antagonist group, suggesting that blocking CB1 signalling may be beneficial in lowering inflammation-induced metabolic abnormalities [104]. It was further found that CB1 receptor-deficient mice did not exhibit LPS-induced fever and that the pharmacological blockade of the CB1 receptor in normal mice also blocked LPS-induced fever. Also, in the liver and spleen of CB1 receptor-deficient mice, a significant reduction in TLR4 mRNA and macrophage nonresponse to LPS were observed, suggesting that the CB1 receptor mediates the LPS-induced inflammatory response through the inhibition of TLR4 [105].

The administration of LPS to pregnant mice revealed increased CB1 receptor protein levels in the uterus. Thus, LPS may activate the ECS by increasing CB1 expression [106]. Muccioli’s study [98] further validates that LPS may be a potent stimulus for endogenous cannabinoid synthesis. By comparing the effects of CB1 and LPS in adipose tissue on adipocyte differentiation and production, it was found that the activation of CB1 receptors increased adipogenesis markers, and LPS decreased the markers of adipocyte differentiation in adipose tissue from wild-type mice. In addition, other studies have shown that LPS counteract the adipogenic effects of CB1 and significantly increase CB1 mRNA expression [107].

Bahrami’s [126] use of a CB1 antagonist in mice with high-fat diet-induced obesity resulted in improved intestinal permeability and inflammation and reduced plasma LPS levels. Moreover, 16S rRNA macro genome sequencing revealed that the CB1 blockade significantly increased the relative abundance of *Akkermansia muciniphila* and decreased Lanchnospiraceae and Erysipelotrichaceae in the intestine. Comparing the gene expression and lipid profiles of the endogenous cannabinoid system in different intestinal segments of germ-free (GF) and conventionally reared (CR) mice, GF mice showed age-dependent modifications in the gene expression and lipid mediator levels of the endogenous cannabinoid system in the intestine. However, this change could be reversed by faecal transplantation [108]. Notably, the intestinal bacterial flora of GF mice was very similar to that of CR mice following caecal transplantation. This deepens the association between gut flora and the ECS. Suriano [127] studied the characterisation of lipid oxidation associated with the ECS in the gut of congenitally obese mice and similarly concluded that the intestinal flora is involved in ECS signalling molecules. After feeding mice a high-fat, high-sugar diet, the relative abundance of *Adlercreutzia*, *Barnesiella*, and *Parasutterella* in the Gram-negative flora of the ileocaecal region was negatively correlated with AEA levels in the small intestine, and increased levels of AEA and 2-AG were detected in plasma [109]. Cani’s [107] study also confirms that the relative abundance of *Akkermansia muciniphila* decreases when AEA levels increase and that the recovery of this bacterium induced by probiotics simultaneously decreases AEA levels. This suggests an unknown association between changes in the relative abundance of specific genera in the intestinal Gram-negative flora and the concentration of specific ECS mediators in the ileum or plasma. Karwad [128] used metabolic enzyme inhibitors of AEA and 2-AG to cause the aggregation of AEA and 2-AG to investigate the effects of AEA and 2-AG on intestinal epithelial cell permeability. The results showed that the assembly of endogenously produced AEA and 2-AG in the intestinal epithelium resulted in increased CB1 and, finally, increased intestinal permeability. It is suggested that the ECS influences intestinal permeability through the aggregation of endogenous AEA and 2-AG and the activation of CB1, which in turn mediates the regulation of the intestinal flora. The dysregulation of the ECS is associated with developing dyslipidaemia, glucose intolerance, and obesity [110,129]. In summary, the ECS regulates adipogenesis, and LPS control this regulation [130]. The intestinal flora can mediate metabolic endotoxaemia and intestinal permeability through the ECS, affecting obesity.

### 6.2. Apelin–APJ

Apelin is an adipokine and peptide hormone that acts by binding to the angiotensin II protein J receptor (APJ). Apelin is widely distributed in all body organs and can influence the development of inflammation through the MyD88 pathway. It also involves physiological processes such as lipid metabolism and endocrine regulation. It is associated with the development of many chronic diseases, so the Apelin–APJ system can be considered a potential target for treating obesity and inflammation [131]. The treatment of adipose tissue explants with HU-210, a cannabinoid receptor agonist, activated the ECS and significantly reduced Apelin and APJ mRNA expression. However, concomitant treatment with HU-210 and LPS markedly increased the mRNA expression of Apelin and APJ, accompanied by an increase in the inflammatory factors IL-1 and TNF-α [111]. This suggests that the LPS is a critical factor in regulating the ECS and Apelin–APJ system and can regulate lipid metabolism by influencing the Apelin–APJ system. In addition, compared to lean mice, obese mice had a higher abundance of Firmicutes, Proteobacteria, and Fibrobacteres and a lower relative and absolute abundance of *Bacteroidetes* and *Deferribacteres* [111]. Changes in the abundance of specific bacteria lead to increased or decreased Apelin–APJ expression, suggesting a latent relationship between the intestinal flora and the regulation of the Apelin–APJ system. The association between LPS, intestinal flora, and lipid metabolism is further illustrated.

### 6.3. GLP

Glucagon-like peptide (GLP) is a hormone released from enteroendocrine L cells. Among other things, GLP-1 inhibits inflammation and promotes mucosal integrity [112]. Pro-inflammatory cytokines such as TNF-α, IL-1β, and IL-6 and the inflammatory biomarker C-reactive protein are all down-regulated by inhibitors of GLP-1 analogues (Liraglutide) and DPP-4, a GLP-1 catabolic enzyme. The inhibition of GLP-1 degradation also improves survival in lipopolysaccharide-induced endotoxaemia [113]. Plasma GLP-1 concentrations increased rapidly after LPS injection in normal mice but not in TLR4-deficient mice. No effect was observed when LPS were given orally to mice, whereas a very rapid secretion of GLP-1 was observed when the intestine was compromised [114]. Lijuan Wang’s [132] experiments also support the idea that LPS affect plasma GLP-1 content via toll-like receptor 4 and that GLP-1 content may reflect intestinal damage to some extent. New research has recently suggested that microbially induced GLP-1 production is not only mediated by LPS and TLR4 but that the activation of other toll-like receptors also leads to increased GLP-1 production [133]. Kahles’ [115] study found that LPS dose- and time-dependently increased total serum GLP-1 concentrations in mice, reducing insulin secretion and blood glucose under inflammatory conditions. In contrast, administering LPS to interleukin-6 knockout mice showed that the elimination of IL-6 significantly attenuated the LPS-dependent GLP-1 concentration and eliminated the increase in serum insulin while partially preventing the decrease in glucose. This indicates that the inflammatory factor IL-6 is essential for LPS-induced GLP-1 production. The GLP-1 agonist (exendin-4) markedly inhibited the inflammatory effects induced by LPS in macrophages and reduced the expression of inflammatory mediators and pro-inflammatory cytokines TNF-α, IL-1β, and IL-6 in macrophages [134]. Notably, exendin-4 inhibited not only the LPS-mediated activation of JNK and AP-1 channels but also nuclear p65 accumulation and transfected NF-κB promoter activity, suggesting that GLP-1 may reduce LPS-induced inflammatory damage primarily through the inhibition of NF-κB channels. It is hypothesised that LPS trigger inflammation and mediate GLP-1 secretion via a MyD88-dependent pathway. However, fewer studies have been conducted on the non-MYD88-dependent path of LPS, so the possibility of influencing GLP-1 secretion via this inflammatory pathway cannot be ruled out.

Obesity affects the metabolism of glucose [135]. The accumulation or antagonistic effects of GLP-1 can significantly alter the outcome of LPS on glucose metabolism. For example, they increased GLP-1 accumulation by oral glucose before LPS injection amplifies the LPS-mediated increase in GSIS (glucose-stimulated insulin secretion). The effect of LPS on blood glucose fluctuations can be blocked with Ex-9 (an antagonist of the GLP-1 receptor) [136]. This all confirms the importance of GLP-1 as a transducer of LPS-mediated glycaemic responses.

Intestinal flora can enhance insulin sensitivity by regulating GLP-1 secretion. The treatment of C57BL/6J mice with antibiotics (vancomycin and bacteriophage) in drinking water before diet-induced obesity (DIO) resulted in a significant reduction in the thick-walled and bacteriophage gates and an increase in GLP-1 secretion in their intestines. Systemic glucose intolerance, hyperinsulinaemia, and insulin resistance were improved in DIO compared to untreated DIO controls [137]. Similar results were obtained in the Wichmann [138] study: the analysis of plasma GLP-1 levels in fasted germ-free (GF) and conventionally reared mice showed that GLP-1 levels were three times higher in the absence of intestinal flora. Subsequently, the use of *E. coli* and Bacteroides thetaiotaomicron, a Gram-negative bacterium that ferments a variety of plant polysaccharides, colonised GF mice and found a reduction in GLP-1 antibodies in both mice colonised with the bacteria compared to GF mice. This all suggests that the gut flora is involved in regulating GLP-1. *A. muciniphila* has been shown to regulate obesity and inflammation. It has been reported that *A. muciniphila* secretes an 84 kDa protein named P9, which interacts with intercellular adhesion molecule 2 (ICAM-2) and directly stimulates L-cells to induce GLP-1 secretion. In addition, P9 stimulates the secretion of IL-6 by macrophages and intestinal epithelial cells to further promote GLP-1 secretion [139,140]. A double-masked, randomised trial [141] confirmed that the administration of probiotics (Lactobacillus roxellanae controlled-release capsules twice daily) increased the plasma levels of GLP-1 and GLP-2 and improved glucose-stimulated insulin levels. Glucagon-like peptide 2 (GLP-2) also reduces the increase in intestinal permeability and prevents the associated loss of barrier function caused by LPS [116,117]. One study [142] found that GLP-2 reduced intestinal injury by reducing inflammatory cytokine secretion and caspase-3 activity and increasing the villus height/crypt depth ratio. It has also been suggested that GLP-2 protects the gut by preventing the down-regulation of multidrug resistance-associated protein 2 (Mrp2) and P-glycoprotein (P-gp) expression, as well as counteracting the increase in the inflammatory factor IL-1β and oxidative stress [118]. Furthermore, the selective alteration of gut microbes and increased endogenous GLP-2 production contribute to improving intestinal barrier function in obesity and diabetes [119]. In conclusion, in the context of inflammation, GLP establishes a novel link between obesity and intestinal flora.

### 6.4. GPR120

G protein-coupled receptor 120 (GPR120) is distributed in the body’s fat and liver. GPR120 affects the expression of glucagon-like peptides and also has a role in regulating adipogenesis, anti-inflammation, and insulin sensitisation [143,144,145]. GPR120 activation inhibits LPS-induced inflammation. gPR120 inhibits NF-κB and MAPK pathways by causing TAK1 inactivation, reducing LPS-induced inflammatory damage [146,147]. GRP120 is a protein that binds to omega-3 fatty acids [148]. One study [149] used ginsenoside Rb2 to explore the effects on LPS-induced inflammation. The results showed that LPS-induced macrophage inflammation was inhibited because of the increased GPR120 expression, which binds more ω-3 fatty acids, and that this enhancing effect of Rb2 must be dependent on GPR120 activation. This also confirms a suppressive effect of GPR120 expression on LPS-induced inflammation. Other studies [150] investigating the involvement of docosahexaenoic acid (DHA) and arachidonic acid (AA) in inflammation have found that DHA and AA attenuate LPS-induced Kupffer cell scorching by promoting the interaction of GPR120 and NLRP3 (NOD-like receptor pyridine-containing structural domain protein 3, a protein associated with the initiation of apoptosis). Liu Yang [120], in assessing the antidiabetic effects of the GPR120 agonist LXT34 in diabetic mice and analysing the effects on inflammation in the liver and adipose tissue, also concluded that GPR120 ameliorated LPS-induced inflammation. lXT34 inhibited inflammation mainly through the TAK1-NF-κB/JNK pathway, significantly reducing macrophage infiltration, inflammatory factor expression, and JNK phosphorylation in the liver and adipose tissue. A comparison of adipogenesis, glucose, and energy homeostasis in GPR120-deficient mice and wild-type mice revealed that GPR120-deficient mice consume less energy and develop obesity, glucose intolerance, and a fatty liver when fed a high-fat diet. This implies that GPR120 can prevent diet-induced obesity [151]. Ichimura [151] reconfirmed the critical role of GPR120 for adiposity by assessing GPR120 expression levels in subcutaneous and omental adipose tissue of lean and obese subjects. GPR120 was well expressed in the adipose tissue of lean individuals and significantly increased in the subcutaneous and omental adipose tissue of obese individuals. GPR120 has also been shown to effectively regulate the gastrointestinal secretion of the enteric insulinotropic hormone GLP-1 [148,152]. In addition, intestinal flora can also influence GPR120 gene expression in intestinal cells. Fredborg [153] explored the effect of 12 intestinal bacteria on cellular GPR120 mRNA abundance and found that among them, *Actinobacteria*, *Firmicutes*, *Bacteroidetes*, and *Aspergillus phylum* all increased intestinal Caco-2 cell GPR120 mRNA expression. Bacteroides fragilis, *Lactobacillus reuteri*, and *Faecalibacterium prausnitzii*, Gram-negative bacteria, showed significant differences (*p* < 0.01).

## 7. Conclusions and Prospect

In this review, we describe the structure of LPS, the mechanism by which LPS trigger inflammation. Based on the hypothesis that LPS mediate obesity by affecting the gut flora, evidence is presented for an interaction between LPS, obesity, and the gut flora, where obesity alters the structure of the gut flora and increases LPS levels and where a reduction in LPS reduces fat gain. In conclusion, LPS derived from Gram-negative bacteria have some influence on the structure of the intestinal flora and, at the same time, have an essential contribution to the development of obesity.

The problem of obesity is becoming more and more serious, and LPS are also an inducing factor of many chronic diseases. This study shows that intestinal intervention is a promising goal to treat obesity and reduce the harm of LPS. The role of LPS, intestinal flora, and obesity is not unidirectional but coordinated with each other. Diet, such as choosing low-fat food, using prebiotics, probiotics, and even drugs (rosiglitazone), can reduce fat intake, improve intestinal permeability, and prevent the harm of LPS. Alternatively, metabolic endotoxaemia and obesity induced by diet can be treated by means of faecal bacteria transplantation (e.g., the transplantation of *A. muciniphila*). However, the clinical therapeutic effects of diet management, drug therapy, and faecal bacteria transplantation have individual differences, and there may be problems of poor effects or adverse reactions. Therefore, this paper puts forward four targets related to LPS: the ECS, Apelin–APJ, GLP, and GPR120. In addition, these targets can affect obesity and intestinal flora to some extent. This review provides direction and reference for the clinical treatment of endotoxaemia, provides new research targets for the research and treatment of obesity, arouses public attention to LPS, and promotes the application of these targets in the treatment of different diseases.

It is noteworthy that the search for evidence in the search for a relationship between LPS and the intestinal flora has focused primarily on the hunt for Gram-negative bacteria and has, therefore, somewhat neglected the synergistic effects of Gram-positive bacteria and their impact on the structure and physiological function of the gut. In the future, further research is needed to investigate the relationship between the abundance of Gram-negative bacteria and metabolic endotoxaemia and the mechanisms of how LPS affect the intestinal environment further to improve the understanding of LPS and intestinal flora.

## Figures and Tables

**Table 1:** Effect of LPS on animals.

Table	Experimental Model	Effect	Refs.
CB1	Male Wistar rats	Obesity, Mesenteric, Retroperitoneal fat	[104]
CB1	Wild-type male C57BL/6 mice and Mice lacking CB1 receptor	Fever	[105]
CB1	Virgin BALB/c female mice	Preterm labour	[106]
ECS	C57BL/6J, B6.V-Lep^ob^/J mice. Germ-free Swiss Webster and Myd88^—^//^—^C57BL/6 mice	Gut permeability, Adipogenesis, Gut microbiota	[98]
CB1	Male C57BL/6 J mice	Diet intake, Body weight, Body composition, Inflammation, Gut barrier, Gut microbiota	[107]
ECS	Conventionally Raised and Germ-free C57BL/6NTac mice	Gut microbiota, Lipid mediator levels	[108]
ECS	C57BL/6J male mice	Gut microbiota, Obesity	[109]
ECS	Genetically obese and diabetic mice	Adipose tissues, Gut microbiota, Inflammation	[110]
ECS, Apelin–APJ	Obesity and diabetes leptin resistance (db/db) mice and C57BL/6 mice	Gut microbiota, Inflammation, Fat tissue	[111]
GLP-1	Adult male Sprague Dawley rats	Allodynia, Inflammation, Gut permeability	[112]
GLP-1	C57BL/6J mice	Cardiovascular system, Oxidative stress, Inflammation, Sepsis	[113]
GLP-1	Male C57BL/6J mice	Gut barrier	[114]
GLP-1	C57BL/6 mice	Inflammation, Insulin secretion, Blood glucose	[115]
GLP-2	Male Sprague Dawley rats	Intestinal paracellular permeability	[116]
GLP-2	Rats	Gut permeability	[117]
GLP-2	Adult female Wistar rats	Intestinal structure and morphology, Inflammation, Oxidative stress	[118]
GLP-2	ob/ob mice	Intestinal permeability, Gut microbiota, Inflammation, Obesity	[119]
GPR120	C57BL/KsJ-db/db obese diabetic mice and C57BL/KsJ-db/m mice	Inflammation, Blood glucose	[120]

## Data Availability

Not applicable.
